# Supplementary material for: Shoot-to-root mobile CEPD-like 2 integrates shoot nitrogen status to systemically regulate nitrate uptake in Arabidopsis
Source: Nat Commun. 2020 Jan 31;11:641. doi: 10.1038/s41467-020-14440-8 (PMC6994653; doi:10.1038/s41467-020-14440-8)
Supplement: Supplementary file 1 — Supplementary Information [file 41467_2020_14440_MOESM1_ESM.pdf]

Supplementary Information

Shoot-to-root mobile CEPD-like 2 integrates shoot nitrogen status to systemically regulate nitrate uptake in *Arabidopsis*

Ryosuke Ota, Yuri Ohkubo, Yasuko Yamashita, Mari Ogawa-Ohnishi

& Yoshikatsu Matsubayashi*

Nature Communications (2020)

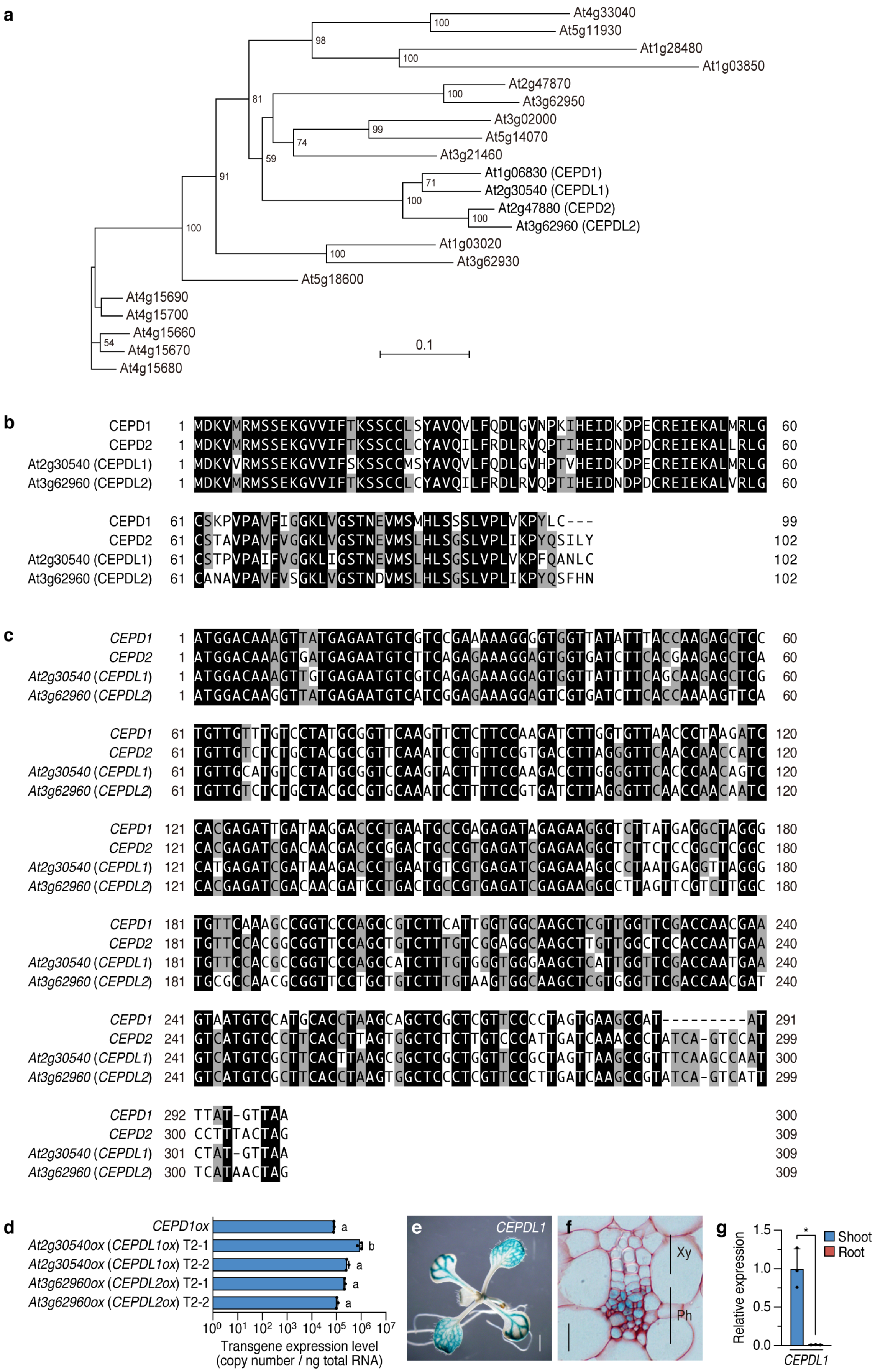

a
At4g33040
At5g11930
At1g28480
At1g03850
At2g47870
At3g62950
At3g02000
At5g14070
At3g21460
At1g06830 (CEPD1)
At2g30540 (CEPDL1)
At2g47880 (CEPD2)
At3g62960 (CEPDL2)
At1g03020
At3g62930
At5g18600
At4g15690
At4g15700
At4g15660
At4g15670
At4g15680
100
98
100
81
100
99
74
59
71
100
100
91
100
100
54
0.1
b
CEPD1 1 MDKVMRMSSEKGVVIFTKSSCCLSYAVQVLFQDLGVNPKIHEIDKDPECREIEKALMRLG 60
CEPD2 1 MDKVMRMSSEKGVVIFTKSSCCLCYAVQILFRDLRVQPTIHEIDNDPDCREIEKALLRLG 60
At2g30540 (CEPDL1) 1 MDKVVRMSSEKGVVIFSKSSCCMSYAVQVLFQDLGVHPTVHEIDKDPECREIEKALMRLG 60
At3g62960 (CEPDL2) 1 MDKVMRMSSEKGVVIFTKSSCCLCYAVQILFRDLRVQPTIHEIDNDPDCREIEKALVRLG 60
CEPD1 61 CSKPVPAVFIGGKLVGSTNEVMSMHLSSSLVPLVKPYLC--- 99
CEPD2 61 CSTAVPAVFVGGKLVGSTNEVMSLHLSGSLVPLIKPYQSILY 102
At2g30540 (CEPDL1) 61 CSTPVPAIFVGGKLIGSTNEVMSLHLSGSLVPLVKPFQANLC 102
At3g62960 (CEPDL2) 61 CANAVPAVFVSGKLVGSTNDVMSLHLSGSLVPLIKPYQSFHN 102
c
CEPD1 1 ATGGACAAAGTTATGAGAATGTCGTCCGAAAAAGGGGTGGTTATATTTACCAAGAGCTCC 60
CEPD2 1 ATGGACAAAGTGATGAGAATGTCTTCAGAGAAAGGAGTGGTGATCTTCACGAAGAGCTCA 60
At2g30540 (CEPDL1) 1 ATGGACAAAGTTGTGAGAATGTCGTCAGAGAAAGGAGTGGTTATTTTCAGCAAGAGCTCG 60
At3g62960 (CEPDL2) 1 ATGGACAAGGTTATGAGAATGTCATCGGAGAAAGGAGTCGTGATCTTCACCAAAAGTTCA 60
CEPD1 61 TGTTGTTTGTCCTATGCGGTTCAAGTTCTCTTCCAAGATCTTGGTGTTAACCCTAAGATC 120
CEPD2 61 TGTTGTCTCTGCTACGCCGTTCAAATCCTGTTCCGTGACCTTAGGGTTCAACCAACCATC 120
At2g30540 (CEPDL1) 61 TGTTGCATGTCCTATGCGGTCCAAGTACTTTTCCAAGACCTTGGGGTTCACCCAACAGTC 120
At3g62960 (CEPDL2) 61 TGTTGTCTCTGCTACGCCGTGCAAATCCTTTTCCGTGATCTTAGGGTTCAACCAACAATC 120
CEPD1 121 CACGAGATTGATAAGGACCCTGAATGCCGAGAGATAGAGAAGGCTCTTATGAGGCTAGGG 180
CEPD2 121 CACGAGATCGACAACGACCCGGACTGCCGTGAGATCGAGAAGGCTCTTCTCCGGCTCGGC 180
At2g30540 (CEPDL1) 121 CATGAGATCGATAAAGACCCTGAATGTCGTGAGATCGAGAAAGCCCTAATGAGGTTAGGG 180
At3g62960 (CEPDL2) 121 CACGAGATCGACAACGATCCTGACTGCCGTGAGATCGAGAAGGCCTTAGTTCGTCTTGGC 180
CEPD1 181 TGTTCAAAGCCGGTCCCAGCCGTCTTCATTGGTGGCAAGCTCGTTGGTTCGACCAACGAA 240
CEPD2 181 TGTTCCACGGCGGTTCCAGCTGTCTTTGTCGGAGGCAAGCTTGTTGGCTCCACCAATGAA 240
At2g30540 (CEPDL1) 181 TGTTCCACGCCGGTCCCAGCCATCTTTGTGGGTGGGAAGCTCATTGGTTCGACCAATGAA 240
At3g62960 (CEPDL2) 181 TGCGCCAACGCGGTTCCTGCTGTCTTTGTAAGTGGCAAGCTCGTGGGTTCGACCAACGAT 240
CEPD1 241 GTAATGTCCATGCACCTAAGCAGCTCGGCTCGTTCCCCTAGTGAAGCCAT---------AT 291
CEPD2 241 GTCATGTCCCTTCACCTTAGTGGCTCTCTTGTCCCATTGATCAAACCCTATCA-GTCCAT 299
At2g30540 (CEPDL1) 241 GTCATGTCGCTTCACTTAAGCGGCTCGCTGGTTCCGCTAGTTAAGCCGTTTCAAGCCAAT 300
At3g62960 (CEPDL2) 241 GTCATGTCGCTTCACCTAAGTGGCTCCCTCGTTCCCTTGATCAAGCCGTATCA-GTCATT 299
CEPD1 292 TTAT-GTTAA 300
CEPD2 300 CCTTTACTAG 309
At2g30540 (CEPDL1) 301 CTAT-GTTAA 309
At3g62960 (CEPDL2) 300 TCATAACTAG 309
d
CEPD1ox a
At2g30540ox (CEPDL1ox) T2-1 b
At2g30540ox (CEPDL1ox) T2-2 a
At3g62960ox (CEPDL2ox) T2-1 a
At3g62960ox (CEPDL2ox) T2-2 a
10^0 10^1 10^2 10^3 10^4 10^5 10^6 10^7
Transgene expression level
(copy number / ng total RNA)
e
CEPDL1
f
Xy
Ph
g
1.5
1.0
0.5
0.0
Relative expression
*
Shoot
Root
CEPDL1

Supplementary Fig. 1 At2g30540 and At3g62960 upregulate the expression of *NRT2.1* when overexpressed. a Phylogenetic tree of the class III glutaredoxin family proteins. Protein sequences were aligned using ClustalW and the tree was generated with the neighbor-joining method. Bootstrap values (above 50%) from 1000 replicates are indicated at each node. **b** Multiple sequence alignment of At2g30540 (CEPDL1), At3g62960 (CEPDL2) and CEPD1/2 polypeptide amino acid sequences. Identical residues are boxed in black, and similar residues are boxed in gray. **c** Nucleotide sequence alignment of *CEPD/CEPDL* family genes. **d** Absolute quantification of transgene transcripts by qRT-PCR analysis of the roots of 10-day-old transgenic plants overexpressing the indicated gene and grown on N-replete medium. Different letters indicate statistically significant differences ($P < 0.05$, two-tailed one-way ANOVA, $n = 3$). **e** Histochemical staining of 10-day-old seedlings transformed with the *CEPDL1pro:GUS* gene. Scale bar = 1 mm. **f** Cross-section of the leaf vascular tissues pictured in 'e'. Scale bar = 10 µm. **g** qRT-PCR analysis of *CEPDL1* transcripts in the shoots and roots of 7-day-old WT plants cultured on 10 mM NO_3^- condition (mean ± SD, *$P < 0.05$ by two-tailed non-paired Student's *t* test, $n = 3$).

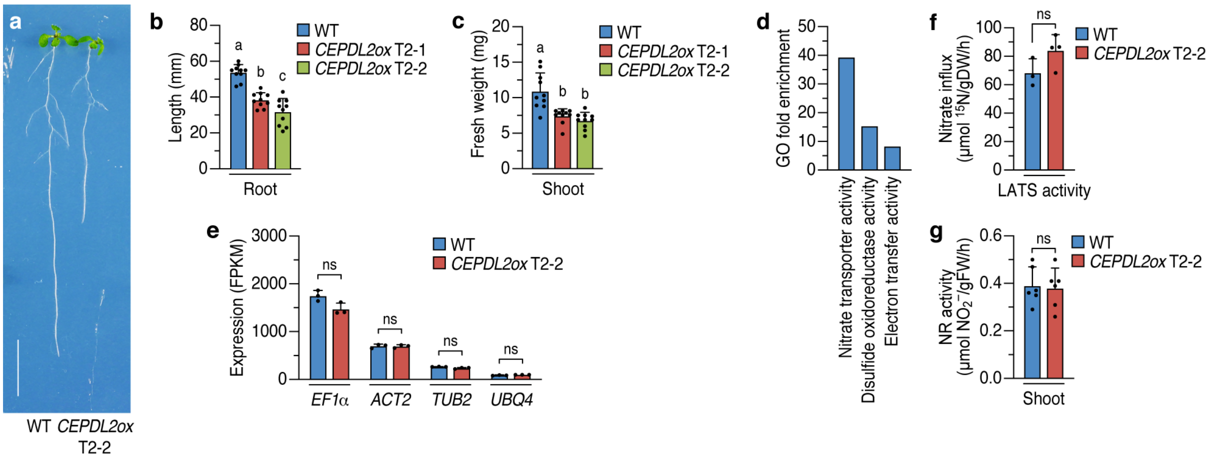

Supplementary Fig. 2 Phenotypes of *CEPDL2ox* plants. a Phenotypes of 10-day-old WT and *CEPDL2ox* plants grown under 10 mM NO_3^- condition. Scale bar = 1 cm. **b** Comparison of root length between 10-day-old WT and *CEPDL2ox* plants (n = 10). **c** Comparison of shoot fresh weight between 14-day-old WT and *CEPDL2ox* plants (n = 10). **d** Gene ontology analysis of the top 150 upregulated genes in *CEPDL2ox* plants. **e** Expression stability of representative reference genes in roots of 10-day-old WT and *CEPDL2ox* plants (n = 3). False discovery rate (FDR) q < 0.05 was considered significant. **f** Calculated LATS activity of the roots of *CEPDL2ox* plants (n = 4). **g** Nitrate reductase (NR) activity in shoots of 17-day-old WT and *CEPDL2ox* plants (n = 6).

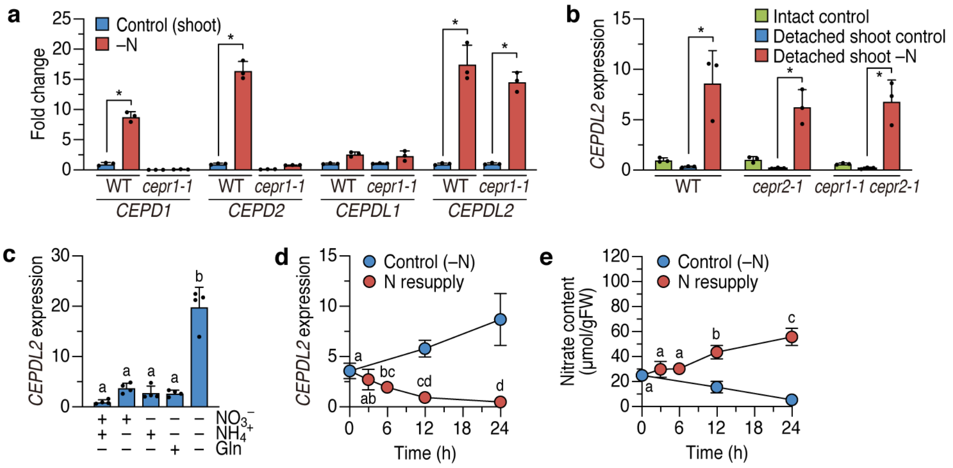

Supplementary Fig. 3 *CEPDL2* expression is regulated by the shoot N status. a qRT-PCR analysis of *CEPD1/2* and *CEPDL1/2* transcripts in the leaves of 12-day-old WT and *cepr1-1* plants after N starvation for 24 h (n = 3). **b** qRT-PCR analysis of *CEPDL2* transcripts in detached leaves of WT, *cepr2-1* and *cepr1-1 cepr2-1* plants after N starvation for 24 h (n = 3). **c** qRT-PCR analysis of *CEPDL2* transcripts in detached WT leaves treated with various N sources for 24 h (n = 3). **d** Changes in *CEPDL2* transcript levels in WT leaves subjected to N starvation for 24 h, followed by continued N starvation (-N) or N resupply (NH_4^+ 10 mM, NO_3^- 10 mM) (n = 3). **e** Changes in nitrate content in WT shoots subjected to N starvation for 24 h, followed by continued N starvation (-N) or N resupply (n = 3).

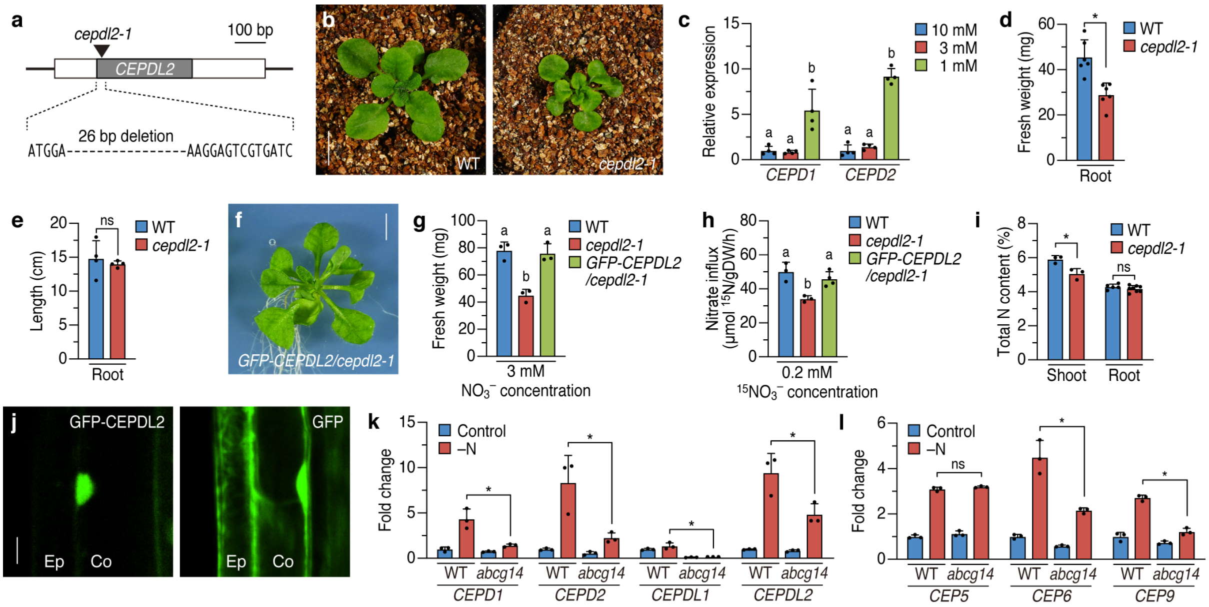

Supplementary Fig. 4 Loss of *CEPDL2* impairs nitrate acquisition. a Schematic representation of the deletion site in *cepdl2-1*. **b** Phenotypes of 4-week-old WT and *cepdl2-1* plants grown in vermiculite with nutrient solutions containing 3 mM NO_3^-. Scale bar = 1 cm. **c** Levels of *CEPD1* and *CEPD2* transcripts in WT shoots grown under various nitrate conditions for 21 d (n = 3). **d** Fresh weight of roots of WT and *cepdl2-1* plants grown under 3 mM NO_3^- conditions for 21 d (n = 6). **e** Root length of WT and *cepdl2-1* plants at 21 d (n = 6). **f** Phenotype of 21-day-old *cepdl2-1* mutant plants complemented with *GFP-CEPDL2* under 3 mM NO_3^- conditions. Scale bar = 5 mm. **g** Fresh weight of shoots of 21-day-old *cepdl2-1* plants complemented with the *GFP-CEPDL2* (n = 3). **h** Recovery of HATS activity in complemented plants. **i** Total N content in shoots and roots of WT and *cepdl2-1* plants grown under 3 mM NO_3^- condition for 21 d. **j** Comparison of GFP signals in the root cortex region of 9-day-old plants expressing GFP-CEPDL2 under the *CEPDL2* promoter or free GFP under the *NRT2.1* promoter. Scale bar = 10 µm. **k** qRT-PCR analysis of CEPD1/2 and CEPDL1/2 transcripts in the shoots of 14-day-old WT and *abcg14* plants after N starvation for 24 h (n = 3). **l** qRT-PCR analysis of *CEP* transcripts in the roots of 14-day-old WT and *abcg14* plants after N starvation for 24 h (n = 3).

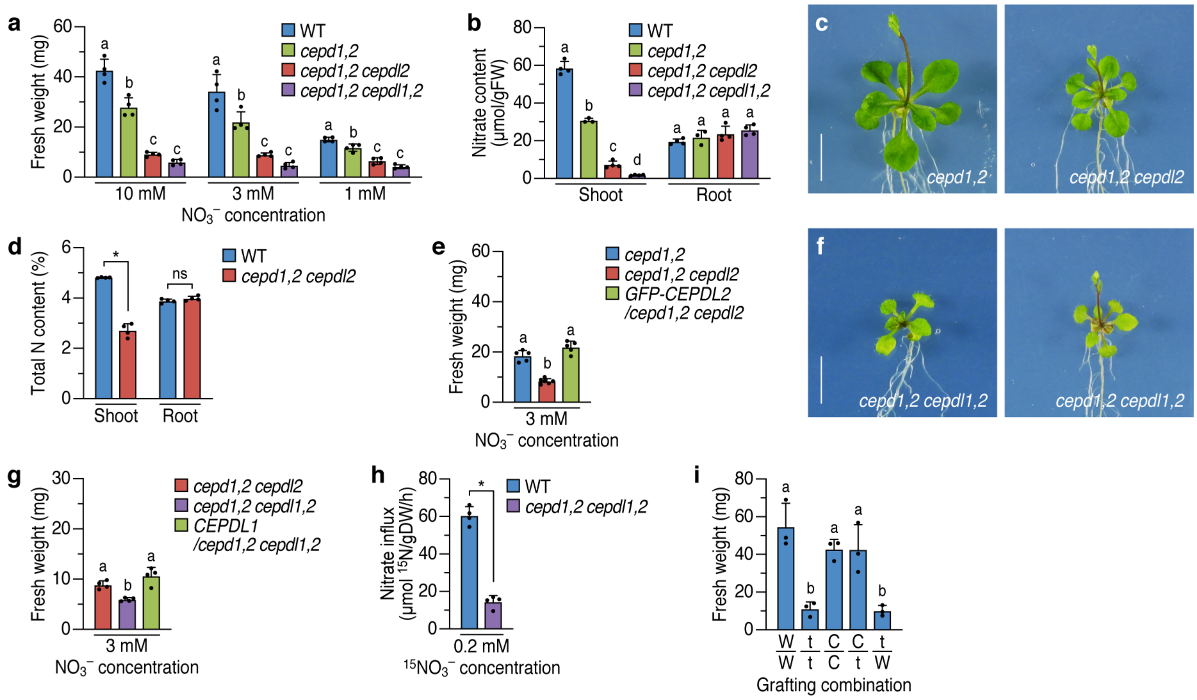

Supplementary Fig. 5 The CEPDL2 and CEPD1/2 systemically regulate root nitrate acquisition. **a** Fresh weight of shoots of WT and multiple-mutant plants grown under various NO_3^- conditions for 17 d (n = 4). **b** Nitrate content in shoots and roots of WT and mutant plants grown under 3 mM NO_3^- conditions (n = 4). **c** Early flowering phenotypes of 21-day-old multiple-mutant plants grown on 3 mM NO_3^- medium. Scale bar = 5 mm. **d** Total N content in shoots and roots of WT and *cepd1,2 cepdl2* triple-mutant plants grown under 3 mM NO_3^- conditions for 17 d (n = 4). **e** Fresh weight of shoots of 17-day-old *cepd1,2 cepdl2* plants complemented with the *GFP-CEPDL2* construct (n = 5). **f** Phenotype of 17-day-old *cepd1/2 cepdl1/2* quadruple mutant plants grown on 3 mM NO_3^- medium (left). Early flowering phenotype of 21-day-old quadruple-mutant plants (right). Scale bar = 5 mm. **g** Fresh weight of shoots of 17-day-old *cepd1/2 cepdl1/2* quadruple-mutant plants complemented with the *CEPDL1* genomic fragment (n = 4). **h** HATS activity in *cepd1/2 cepdl1/2* quadruple-mutant roots (n = 4). **i** Fresh weight of shoots of reciprocally grafted plants (n = 3). Abbreviations: W, wild-type; t, *cepd1,2 cepdl2* triple mutant; C, *cepd1,2 cepdl2* triple mutant complemented with *GFP-CEPDL2*.
