# Supplementary material for: Shoot-to-root mobile CEPD-like 2 integrates shoot nitrogen status to systemically regulate nitrate uptake in Arabidopsis
Source: Nat Commun. 2020 Jan 31;11:641. doi: 10.1038/s41467-020-14440-8 (PMC6994653; doi:10.1038/s41467-020-14440-8)
Supplement: Supplementary file 3 — Description of Additional Supplementary Files [file 41467_2020_14440_MOESM3_ESM.pdf]

Description of Additional Supplementary Files

File Name: Supplementary Data 1
Description: List of differentially expressed genes in roots of CEPDL2ox plants identified by RNA-Seq, sorted by Log2-fold change. Genes with a q-value < 0.05 and absolute log2 fold change > 1 were defined as differentially expressed genes.

File Name: Supplementary Data 2
Description: Primer and probe sequences used in this study.
